# Pain priors, polyeidism, and predictive power: a preliminary investigation into individual differences in ordinary thought about pain

**DOI:** 10.1007/s11017-021-09552-1

**Published:** 2021-12-17

**Authors:** Emma Borg, Sarah A. Fisher, Nat Hansen, Richard Harrison, Deepak Ravindran, Tim V. Salomons, Harriet Wilkinson

**Affiliations:** 1 University of Reading, Reading, UK; 2 University of Vienna, Vienna, Austria; 3 Royal Berkshire NHS Foundation Trust, Reading, UK; 4 Queen’s University, Kingston, ON Canada

**Keywords:** Mental view of pain, Bodily view of pain, Polyeidic view of pain, Chronic pain, Psychological interventions for pain management

## Abstract

According to standard philosophical and clinical understandings, pain is an essentially mental phenomenon (typically, a kind of conscious experience). In a challenge to this standard conception, a recent burst of empirical work in experimental philosophy, such as that by Justin Sytsma and Kevin Reuter, purports to show that people ordinarily conceive of pain as an essentially bodily phenomenon—specifically, a quality of bodily disturbance. In response to this bodily view, other recent experimental studies have provided evidence that the ordinary (‘folk’) concept of pain is more complex than previously assumed: rather than tracking only bodily or only mental aspects of pain, the ordinary concept of pain can actually track either of these aspects. The *polyeidic* (or ‘many ideas’) analysis of the folk concept of pain, as proposed by Emma Borg et al., captures this complexity. Whereas previous empirical support for the polyeidic view has focused on the context-sensitivity of the folk concept of pain, here we discuss individual differences in people’s ‘pain priors’—namely, their standing tendencies to think of pain in relatively mind-centric or body-centric ways. We describe a preliminary empirical study and present a small number of findings, which will be explored further in future work. The results we discuss are part of a larger programme of work which seeks to integrate philosophical pain research into clinical practice. For example, we hypothesise that variations in how patients with chronic pain are thinking about pain could help predict their responses to treatment.

## Introduction

There seem to be different ways of thinking and talking about pain. Sometimes people focus on mental aspects, as when pain is said to be something that feels a certain way; and other times people focus on bodily aspects, as when pain is said to be located in a certain part of the body. The first way of thinking (which we will explore further in the following section below) accords with the standard understanding of pain among philosophers—Saul Kripke, for example, analyses pain as a kind of feeling [1], while Michael Tye has argued that pains are representational mental states [2]. In clinical settings, too, pain can be conceived of in mental terms. For example, the International Association for the Study of Pain defines pain as ‘An unpleasant sensory and emotional experience associated with, or resembling that associated with, actual or potential tissue damage’, stating in an accompanying note that pain is ‘always a personal experience’ [3].^1^ Recently, though, a group of experimental philosophers have argued that the second, bodily way of thinking about pain accords with the ordinary ‘folk’ concept, meaning that lay people distinguish between the existence of pain and the feeling of pain [4–7]. Pain itself, on this view, exists always and only as a bodily property; it is merely the *feeling* of pain that qualifies as a mental state (the bodily view is discussed further below; see ‘Pains as bodily properties’). If this claim is correct, it would reveal an important discontinuity between ordinary thought (and talk) about pain and philosophical orthodoxy, which has otherwise been assumed to enjoy considerable intuitive appeal.^2^ Elsewhere, we have argued that the experimental evidence for a purely bodily ordinary concept of pain is unconvincing, and that what is needed is a more complex view of the ordinary concept of pain—one which is able to accommodate both mind-centric and body-centric ways of thinking [10, 11]. This more complex analysis of the folk concept of pain, dubbed the *polyeidic* (or ‘many ideas’) view, is empirically supported by a series of recent experiments reported by Tim Salomons et al. [12].^3^ In this work, participants were given a series of vignettes where the physical conditions for attributing pain were met but the mental conditions were absent. So, for instance, participants were asked to consider an ice skater who had severely damaged her wrist, where this damage was evident to all but where the skater herself said that the injury did not hurt. Participants were then asked to judge whether the subjects in these vignettes ‘had pain’. Where the mental component (feeling pain) was absent, participants tended to judge that the subject did not have pain, showing that, at least in some contexts, participants appeared to operate with a mind-centric conception of pain.

We should note, however, that the empirical evidence does not conclusively refute either the bodily view or the mind-centric view, for rejoinders remain available to proponents of each stance. For one, it might be argued that the surface form of ordinary talk about pain may not be a good guide to its underlying meaning. In this way, proponents of the bodily view might argue that utterances that do not fit easily with their account are actually instances of loose talk—for example, they might claim that ‘I have a stabbing pain’ really means *my pain feels a certain kind of way, like I’m being stabbed*. Yet the very same move is available to proponents of the mental view for utterances that do not fit easily with a mental account—for example, they might claim that ‘I have a pain in my leg’ is loose talk for *I have a cause of pain in my leg*. These strategies no doubt require further discussion, but for now we note that the polyeidic view is not driven to reanalyse language in this way (nor is the alternative polysemy-based approach proposed by Michelle Liu [13]). Instead, descriptions of pains can simply be taken at face value. So while proponents of mental and bodily views might seek to avoid challenge by arguing that the surface form of some particular utterance does not reflect its underlying meaning, for the remainder of the paper we will set this response to the side. In what follows, then, when people are content to describe pain as a feeling, we will assume this shows that they are thinking of pain as a feeling (not merely engaging in loose talk); and when they are content to describe pain as a bodily phenomenon, we will assume that this shows they are thinking of pain as a bodily phenomenon.^4^

The earlier empirical studies by Salomons et al. focus on *contextual variation* in people’s views about pain—that is, their ability to adopt a more mind-centric or more body-centric view depending on the scenario presented to them—but an important remaining question is whether there are also *individual differences* in people’s views of pain. That is to say, do some people tend towards a more mind-centric view of pain and others towards a more body-centric view (independently of the particular situation they are faced with)? And are these tendencies, or ‘pain priors’, more or less important than contextual effects on their judgments?^5^

These are important questions because their answers could have clinical repercussions. We are especially interested in the treatment of chronic pain patients, including the degree to which these patients are able to benefit from psychological pain management techniques. The way patients are thinking of pain (as more bodily or more mental), we suggest, may affect the extent to which they think of their pain as something that can be managed or improved by changing patterns of thought and behaviour, as opposed to thinking of it as something that can be ameliorated only through, say, surgical interventions.

In this paper, we report some preliminary observations on data gathered in the course of beginning to investigate differences in individuals’ ‘pain priors’—namely, their tendencies to think of pain in a way that is either relatively mind-centric or relatively body-centric. The structure of the paper is as follows: The next two sections explore the mental conception of pain and the bodily conception of pain, respectively. In both discussions, we indicate the kinds of materials that we think could be successfully used to probe subjects’ views. The subsequent section summarises the differences between the mental and bodily conceptions of pain and briefly introduces the pluralistic polyeidic view. There follow selected findings from our preliminary empirical study, which represents an initial attempt to investigate differences in individuals’ pain priors as distinct from contextual effects. The final section sets out why an investigation of both factors—contextual variation and individual differences—is important, outlining the clinical role these factors might play.

## Pains as mental properties

The first view we consider assumes that people ordinarily conceive of pains as being mental states or properties—albeit ones that are typically occasioned by disturbances in body parts. In principle, such states or properties need not always be part of our conscious experience, given that some of our mental life is unconscious*.* However, if pains could be unconscious, that would raise the question of what distinguishes mental states that are painful from those that are not, if not the way they feel. There are various possible answers that might be given here—for example, painful states might be thought of as those which have certain functional profiles [14], representational contents [15], or neural signatures [16]. However, more commonly, pain is simply analysed as a conscious mental phenomenon (e.g., [1]). For current purposes, then, we focus on the proposal that people ordinarily conceive of pains as feelings of a particular kind. This understanding of pains as feelings is what we mean when we talk about the ‘mental conception’ of pain.^6^

As discussed by Emma Borg et al. [10, pp. 36–37], simply asking individuals directly whether they believe that pain is ‘in the mind’ is unlikely to elicit a response that reveals whether or not they are thinking of pain in mental terms—due, at least in part, to the fact that this way of speaking is commonly heard as insinuating that the pain is not real. A more appropriate strategy, then, and one which is commonly used in the experimental literature, is to probe intuitions about a cluster of more specific claims. By teasing apart the monolithic claim that ‘pain is a mental state’ into more nuanced subclaims, we both operationalise the claim, making it possible to ask subjects for their views more directly, and provide a framework able to capture the potential for individuals’ views to pattern differently across the more specific subclaims (discussed further in our pilot study below). For these reasons, we break down the mental conception of pain into six component subclaims, organising them in rough order of centrality to the overarching view (while noting that they are not necessarily exhaustive of it). We begin with the core definitional claim:

- (A1) Pains are conscious mental experiences.

As noted above, it is theoretically possible to hold a broadly mental conception while denying (A1) insofar as one believes that pains can be unconscious mental states. However, we suspect that ordinarily people do not tend to make this distinction; instead, where they are inclined to adopt a mental perspective, we speculate that they are likely to think of pain simply as a kind of feeling, as per the narrower mental conception of pain we are concerned with here. Thus we take (A1) to be central to a common-sense mind-centric way of thinking about pain. It may be possible to probe whether people agree with (A1) by asking them to assess the truth of statements like ‘Pain is part of how we experience things that happen in the body’ or ‘The International Association for the Study of Pain is right to define pain as “always a psychological state”, even if it is caused by an injury’. The definitional claim in (A1) entails the following necessity and sufficiency claims:

- (A2) Feeling pain is necessary for pain to exist.
- (A3) Having a feeling of pain is sufficient for pain to exist.

Those who adopt a purely mental conception of pain, whereby pains are feelings, should endorse (A2), denying that pains can ever exist unfelt. Thus, they should agree with a statement like ‘All pains hurt’.^7^ Equally, those who think that having pain is feeling pain should endorse (A3).^8^

On the flipside, someone operating with a purely mental conception of pain would not consider bodily damage to be necessary for pain.

- (A4) Bodily damage is not necessary for pain to exist.

According to the mental conception of pain, someone could have pain despite having suffered no damage at the relevant bodily location. Those who are operating with a mental conception of pain, then, should disagree with a statement like ‘If we feel pain, some part of our body must be damaged’. Alternatively, they might agree with a statement like ‘Although pain evolved to warn us about bodily damage, sometimes pain doesn’t perform any useful role in informing us about the state of our bodies’. As discussed below, we believe endorsement of (A4) may have important clinical ramifications.

The mental conception of pain similarly denies that bodily damage is sufficient for pain.

- (A5) Bodily damage is not sufficient for pain to exist.

In other words, it is thought to be possible, at least in principle, for someone to suffer bodily damage without having pain. Those who are operating with a mental conception of pain, then, should disagree with a blanket statement like ‘Pain does not go away until the injury that causes it goes away’. Alternatively, they might agree with a statement like ‘It is possible to overcome pain mentally’. Again, the potential clinical implications of endorsing (A5) will be discussed below.

Finally, the mental conception of pain denies that the amount of pain someone has is determined entirely by the amount of bodily damage suffered.

- (A6) The amount of pain is not fixed by the amount of bodily damage.

If pains are experiential, they may dissociate from the injury that causes them. Therefore, it will not necessarily follow that greater injury means greater pain, or that lesser injury means lesser pain—although this might usually be the case). Instead, a range of other factors—such as one’s pain threshold, general psychological state, or views on the meaning or usefulness of one’s pain—could intervene to modulate the amount of pain that exists. Whether or not an individual endorses (A6) could perhaps be confirmed by eliciting agreement with a statement like ‘Thoughts and feelings can influence the amount of pain someone has’ or eliciting disagreement with a statement like ‘More tissue damage always equals more pain’.^9^^,^ 10

To summarise, it is not, we contend, appropriate to probe whether people are thinking of pain in a mind-centric way by asking them a direct question such as ‘Do you think X’s pain is in X’s mind?’, since they may well expect a positive answer to this kind of question to be heard as expressing a pejorative claim about the reality of X’s pain. Instead, it is better to probe a subject’s agreement with ancillary claims, the truth of which depends on the acceptance of a mind-centric view—for instance, by examining whether subjects agree with a claim like ‘Pain is always a psychological state, even if it is typically caused by bodily damage’. Having considered how one might explore the mind-centric perspective, we now turn to the bodily view.

## Pains as bodily properties

In contrast to the mental conception, the bodily conception takes pains to be properties of physical disturbances in the body (see [4–7]). Presumably, not *all* bodily disturbances are taken to be painful; itches and tickles, for example, appear to be forms of bodily disturbance that are not painful. We assume, then, that someone who is operating with a bodily conception of pain is thinking of pains as properties of bodily damage or injury (leaving open exactly which disturbances qualify as such). The idea is that just as one might intuitively ascribe a range of properties to other physical phenomena—size, colour, temperature, loudness, and so on—so too is the property of pain ascribed to the physical phenomenon of bodily injury.

It is important to note that the bodily conception of pain, as we understand it here, locates pain at (typically peripheral) bodily locations where injury has occurred. So, for example, pains are thought to be properties of stubbed toes, cut fingers, sunburnt noses, and so on.^11^ A different view might allow for pains to be located elsewhere in the body, such as in a malfunctioning nervous system or a disturbed neural pathway.^12^ Although such a view might be considered a ‘bodily’ conception of pain, broadly construed, it is not our target here. Instead, when we talk about the bodily conception of pain, we confine our attention to the classic version of the view, as elaborated by Kevin Reuter and Justin Sytsma [4–7].

As above, we identify six key subclaims associated with the bodily conception of pain, listed roughly in order of centrality (but, again, with the caveat that these claims are not necessarily exhaustive of the view). First is a definitional claim, which contrasts with (A1) above:

- (B1) Pains are properties of bodily damage.

Endorsement of this claim might be probed directly by asking people to assess the truth of a statement like the following: ‘When someone says “I am in pain” they are talking about a property of their body’.

The bodily conception denies the necessity and sufficiency claims associated with the mental conception in (A2) and (A3) above, generating the following negative claims:

- (B2) Feeling pain is not necessary for pain to exist.
- (B3) Having a feeling of pain is not sufficient for pain to exist.

Those operating with a bodily conception, then, should allow for the possibility of unfelt pain; and they should agree with a statement like the following: ‘When someone gets distracted from pain, the pain is still there, the person just doesn’t notice it’.^13^ Moreover, having a feeling of pain is not considered to be sufficient for having pain on the bodily conception, contrary to (A3) above.^14^ Thus, endorsement of (B3) predicts agreement with a statement like ‘It is possible that someone can be wrong when they feel like they are in pain’.

Conversely, the bodily conception is committed to the necessity claim the mental conception rejects, as per (A4) above. In other words, the claim in (B1) entails the following:

- (B4) Bodily damage is necessary for pain to exist.

According to the bodily conception, pain arises only with bodily damage; therefore, it cannot arise in the absence of bodily damage. Put simply: there is no pain without injury. Someone operating with this conception of pain, then, should agree with a statement like ‘If we feel pain, some part of our body must be damaged’. As discussed below, we believe endorsement of (B4) could have important clinical implications.

The bodily conception of pain also appears to generate the following sufficiency claim, which is denied under the mental conception in (A5) above:

- (B5) Bodily damage is sufficient for pain to exist.

According to (B5), pain always exists when there is bodily damage—regardless of how the subject feels. Strictly speaking, the bodily conception of pain need not be committed to this claim. However, rejecting it would immediately raise the question of what distinguishes bodily damage that is painful from bodily damage that is not. Since it is unclear how those operating with the bodily conception might respond here—obviously, they could not appeal to any mental criteria—we will assume that they do, in fact, endorse the positive sufficiency claim in (B5). Endorsement of (B5) would imply agreement with the following vignette, for example: ‘Melinda and Mary-Lou are conjoined twins who share a leg. They stub a toe on the foot of the shared leg. Melinda says “ouch”, Mary-Lou doesn’t seem to notice the stubbed toe. In this case, both twins have pain.’ And it may lead to disagreement with a statement like ‘It is possible to overcome pain mentally’. After all, if pain were an inevitable concomitant of bodily injury, it would presumably not be the kind of thing that is influenced by how individuals think or feel about it.^15^ Again, we believe the extent to which individuals subscribe to (B5) could have important clinical implications, which we return to below.

It is less clear whether or not the bodily conception of pain is committed to the following claim about amount of pain, which is the contradictory of (A6) above:

- (B6) The amount of pain is fixed by the amount of bodily damage.

We will therefore treat (B6) as being less central to the bodily view than the other five subclaims laid out above.

Having set out the kinds of probes we consider appropriate for investigating whether a subject is operating with a mental or a bodily conception of pain, we turn now to the polyeidic view, which denies that the ordinary concept of pain is either purely mental or purely bodily.

## The polyeidic view

Summarising the discussion of the last two sections, Table 1 shows how the mental and bodily conceptions of pain discussed in the philosophical literature vary across a series of key claims.

**Table 1:** Key claims of mental and bodily conceptions of pain

Statement		Mental	Bodily
1a	Pains are conscious mental experiences	True	False
1b	Pains are properties of bodily damage	False	True
2	Feeling pain is necessary for pain to exist (no unfelt pain)	True	False
3	Having a feeling of pain is sufficient for pain to exist (no illusory pain)	True	False
4	Bodily damage is necessary for pain to exist (no pain without injury)	False	True
5	Bodily damage is sufficient for pain to exist (no injury without pain)	False	True
6	The amount of pain is fixed by the amount of bodily damage	False	Unclear

According to the pluralistic approach of the polyeidic view, pain can in fact be conceptualised in each of these ways (and potentially in various other ways too) [10]. Thus, the pure mental and pure bodily views described above can be thought of as two extremes lying at either end of a spectrum which captures ordinary thought and talk about pain, where this is sometimes relatively mind-centric and other times relatively body-centric. Evidence for this polyeidic view comes from a series of vignette-based studies [12] which found that participants tended to adopt a mind-centric perspective in response to some scenarios, while in response to others they tended to think about pain in a bodily way. The results suggest that the folk concept of pain may be more complex and situationally variable than either a purely mental or a purely bodily conception allows.

A question raised by these studies, however, is whether the way in which someone thinks about pain is purely a function of the scenario given or whether individuals also have varying ‘pain priors’—that is, pre-existing tendencies to think about pain in relatively mind-centric or body-centric ways. Although the polyeidic view takes the folk concept of pain to be malleable across contexts, it also allows for the possibility that some individuals tend towards a mental conception of pain while others tend towards a bodily conception. As discussed below, determining whether shifts in mental and bodily perspectives are entirely a result of the context of judgment or whether they are also influenced by differences in the way a person generally conceives of pain could have clinical relevance. On the one hand, if shifts in view are entirely conditioned by contextual factors, then it will be extremely important to think carefully about the contexts in which clinician–patient exchanges about pain occur, to ensure that problematic conceptions of pain are not inadvertently promoted by either party. On the other hand, if standing ‘pain priors’ have a role to play, then identifying what these dispositions are could be a valuable part of preliminary work with patients, to ensure they are able to benefit as much as possible from various treatment options. In view of the above, our preliminary empirical study serves as an initial investigation into the potential balance between contextual and individual factors in people’s pain judgments.

## Pilot work on the existence of individual differences

In a pilot study, we presented participants with a series of statements about pain, designed to probe their intuitions about the theoretical claims discussed above.^16^ For each experimental item, participants rated their agreement on a seven-point Likert scale—with 1 labelled ‘Strongly Disagree’ and 7 labelled ‘Strongly Agree’. In analysing the data, we inverted the scores of all items for which responses greater than 4 are indicative of body-centricity. That way, body-centric responses are always marked by scores below 4 and mind-centric responses are always marked by scores above 4, allowing the two alternative views to be plotted on a single scale. Our preliminary findings suggest that at least some individuals do occupy different positions along a mind–body spectrum, although we must stress that these findings remain to be validated with a larger sample.

### Contextual effects

As in the earlier vignette studies by Salomons et al. [12], some experimental items elicited generally mind-centric responses while others elicited generally body-centric responses. An example of an item that elicited a generally mind-centric response is the following:

Thoughts and feelings can influence the amount of pain someone has.

People tended to agree with this statement (*M* = 5.49), suggesting that, in response to this prompt, pain may be thought of as dissociable from bodily damage and dependent, at least in part, on subjective experience.^17^

In contrast, below is an example of an item that elicited a generally body-centric response:

When someone is in pain they can sometimes get so wrapped up in other things (like reading a good book or trying to do a puzzle) that they are not aware of their pain for a time, even though the pain still exists.

People tended to agree with this statement (*M* = 2.86 after inversion), indicating that participants were thinking about pain as dissociable from subjective experience and potentially dependent on bodily damage. In other words, this prompt elicited evidence of bodily thinking about pain, whereas the previous one elicited evidence of thinking about pain in mental terms.^18^ These results support the finding from our earlier studies that individuals are able to conceive of pain in both mental and bodily ways. However, we were interested to explore whether these contextual effects were modulated by individual differences—that is to say, whether people’s pain judgments are a feature not only of the contextual situation they are presented with but also of their standing view of pain.

### Individual differences

The clearest demonstration of individual differences in pain priors would be to find that some individuals consistently gave mind-centric responses (scoring above 4 on every item), while others consistently gave body-centric responses (scoring below 4 on every item). However, this pattern did not emerge clearly from our pilot data. Taking each individual’s mean response, across all sixty items, most participants clustered around the midpoint of 4. This is despite most showing willingness to give ratings towards the extremes of the Likert scale when assessing individual items.

It may be, then, that many people have highly malleable conceptions of pain, whereby they access both mental and bodily aspects of pain fairly flexibly and fluidly depending on precisely what they are responding to.^19^ This finding would perhaps be unsurprising in light of the polyeidic view, which predicts that people are capable of thinking about pain in both mind-centric and body-centric ways. Indeed, our earlier experimental findings suggest that pain judgments are, at least in part, a function of the context in which the judgment is made [12]. Thus, it is to be expected that individuals will shift along the spectrum somewhat when evaluating different statements.

Nevertheless, the results provide early indicative evidence that responses are not driven *only* by situational factors. First, it is notable that a small number of participants appear to have been operating with a relatively mind-centric view or a relatively body-centric view. A handful of individuals had overall mean scores that were above 4.5 (indicating a consistently more mind-centric view), while one or two at the other extreme had overall mean scores below 3.5 (indicating a consistently more body-centric view). If these findings were replicated for a larger sample, this would show that there are some individuals in the general population whose pain judgments are influenced by their prior, standing-state view of pain (alongside contextual influences).

**Fig. 1:**
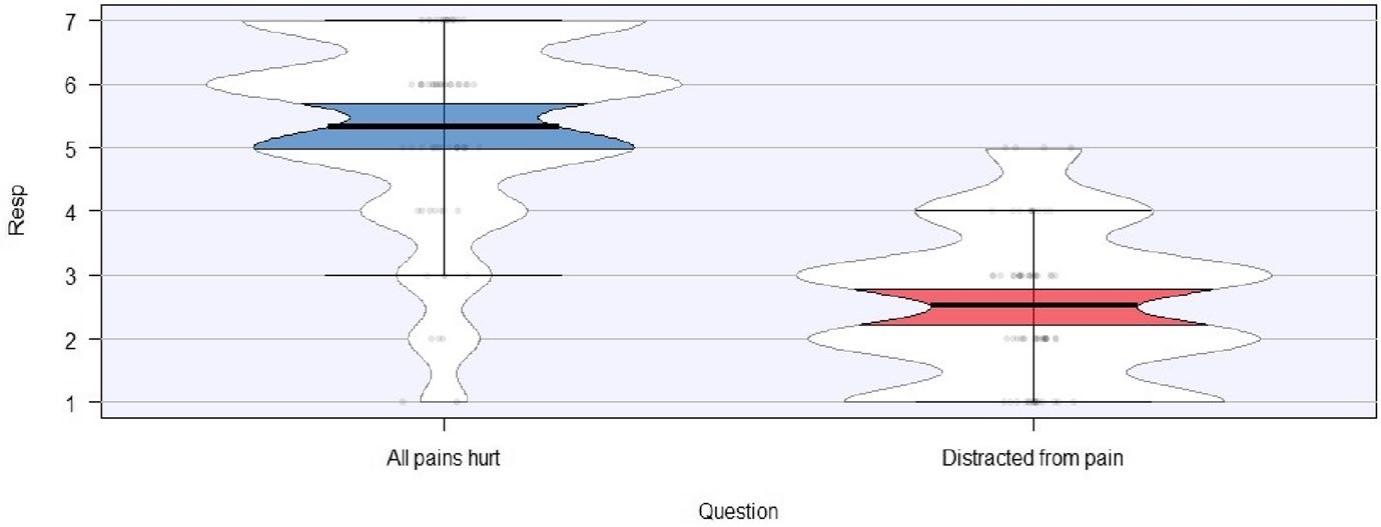
Illustrative probes of the claim that feeling pain is necessary for having pain

Second, we found some range in individual responses with respect to the specific subclaims associated with the mental and bodily conceptions laid out above. In other words, we found early indications that some items may push participants towards either a mind- or body-centric view, while the effect of other items may be less pronounced. So, for instance, recall that one of the claims associated with the mental conception is that feeling pain is necessary for pain to exist. The figure below plots responses to two of the illustrative statements discussed in reference to (A2) and (B2) above (Fig. 1). In our preliminary results, the following statement elicited a mind-centric mean response (*M* = 5.32, *SD* = 1.54):

All pains hurt.^20^

In contrast, the following statement elicited a body-centric mean response (*M* = 2.52, *SD* = 1.16):

When someone gets distracted from pain, the pain is still there, the person just doesn’t notice it.

The apparent reversal in judgments here highlights the force of contextual effects. Different ways of framing the probe can, we believe, elicit different views about whether or not bodily damage is necessary for pain to exist. It may be that, at least with respect to items like the two above, pain priors are playing a minor role in people’s judgments relative to contextual effects. Testing with a larger sample will help to confirm which experimental items and theoretical claims pattern in this way.

**Fig. 2:**
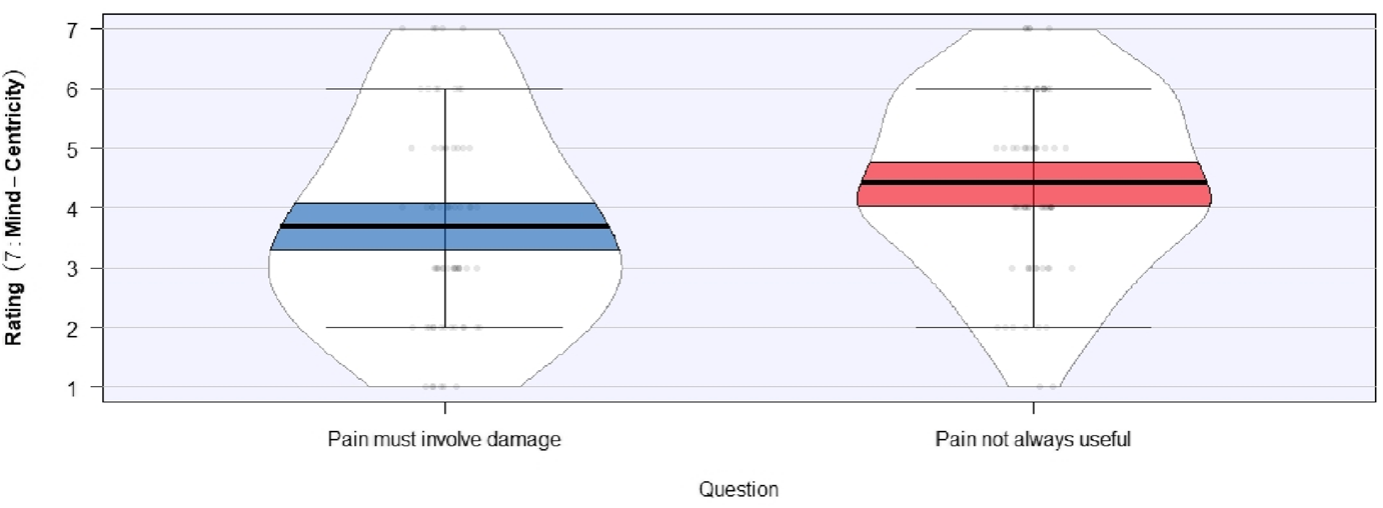
Illustrative probes of the claim that having bodily damage is necessary for having pain

However, some other experimental items yielded more mixed results. So, for instance, turning to the contrasting necessity claim associated with the bodily conception of pain—that bodily damage is necessary for pain to exist—Fig. 2 plots responses to two of the illustrative statements discussed in (A4) and (B4) above. In our preliminary data, the following statement elicited a slightly body-centric mean response (*M* = 3.68, *SD* = 1.69):

If we feel pain, some part of our body must be damaged.

Meanwhile, the following statement elicited a slightly mind-centric mean response (*M* = 4.42, *SD* = 1.50):

Although pain evolved to warn us about bodily damage, sometimes pain doesn’t perform any useful role in informing us about the state of our bodies.

That said, both means remain fairly close to the midpoint, and the more elongated plots represent a larger spread of responses. That there was such a spread of responses, despite the experimental items remaining constant across all participants, suggests that some factor or factors in addition to those generated by the experimental items themselves may be in play. In that case, explaining this pattern would require appealing to something beyond contextual effects. It is too early to conclude with any certainty that this additional effect derives from individuals’ standing views of pain, but differences at this level are a good explanatory candidate. If some people do simply tend to think of pain in a more mental way while others tend towards a more bodily conception, such individual variation might explain divergent answers to prompts like the above pair. Whether or not this is the right hypothesis, though, remains to be shown through testing with a larger sample. Ultimately, although we cannot say definitively whether pain priors play an important role with respect to at least some pain judgments, our preliminary results do indicate that there may be more to the story than just the contextual effects brought about by experimental items.

To conclude, our preliminary results support three findings. First, they are consistent with earlier results showing the existence of contextual effects on pain judgments. Second, they go beyond earlier findings by beginning to demonstrate that contextual effects may be more pronounced with respect to certain kinds of claims about pain than others. Third, they indicate that, for at least some claims about pain, the judgments made by individuals may be influenced by more than just the contextual effects of the prompt. We take these results as preliminary evidence that the search for standing-state elements of an individual’s conception of pain is justified.

## Conclusions and clinical repercussions

We believe that the ways in which people think about pain could have important clinical implications. For one, if people differ in how they are conceptualising pain in a given context, then communication about pain among them may be problematic. As a crude example, if a clinician comes to an exchange with a particularly body-centric conception of pain—focusing on the somatic aspects at the expense of how the pain feels for the patient—while the patient is focused on more affective aspects, or vice versa, their conversations about pain are unlikely to go well (see [11, sec. 4] for further discussion). The pilot study surveyed here forms part of a larger programme of work which seeks to integrate philosophical pain research into clinical practice, demonstrating the relevance of a live debate in experimental philosophy to practical medicine. We are particularly interested in how pain is conceptualised by chronic pain patients—those who report pain lasting for longer than three months [22]—as well as whether the way these patients think about their pain affects their responsiveness to psychological treatment. Currently, in the United Kingdom, there is no standardised method for stratifying chronic pain patients in terms of their likelihood of benefiting from psychological pain management treatments. Furthermore, once they commence treatment, they may be faced with a relatively undifferentiated treatment programme. It is worth exploring whether, at such junctures, outcomes might be improved by establishing how patients are thinking about their pain and tailoring interventions accordingly.

As alluded to earlier, when individuals think of pains as being properties of damage at a particular bodily location, they may find a referral for psychological treatment puzzling. Some patients may be thinking of bodily damage as *necessary* for pain—either because they have a standing tendency to do so or because features of the context encourage them to adopt that view. These patients could then be expected to think of their chronic pain as implying that there is damage at the relevant bodily location. On that basis, the most appropriate intervention from their perspective would involve identifying and rectifying that damage, rather than focusing on thoughts and behaviours. Likewise, patients who are thinking of bodily damage as *sufficient* for pain would presumably reject the possibility that their pain could be reduced or eliminated by changing their thoughts and behaviours. As noted, if pain were purely a concomitant of bodily injury, it would presumably be unaffected by such interventions. We would predict, then, that someone who is thinking about pain in a highly body-centric way is unlikely to consider psychological treatment to be suitable and may fail to engage with it—perhaps refusing the treatment upfront, dropping out during the course, or deriving relatively little benefit from the intervention.

Conversely, patients who are—or become—less attached to the bodily conception of pain, as understood here, may be more open to psychological treatment options. The hypothesis guiding our investigation is that by recognising this, and tailoring information and interventions appropriately, it may be possible to strengthen patients’ therapeutic allegiance to their consultant and, ultimately, improve their ability to manage—or even overcome—their pain. The observations we report here are a first step in exploring this possibility. As such, they bring an extant debate in experimental philosophy closer to bearing on real-world clinical practice.

## Appendix

These items are currently unvalidated for use as a cohesive measure.

The 60 experimental items listed below were presented to participants quasi-randomly, with the instruction: ‘You will now see a series of statements about pain. Using the scale, where 1 = strongly disagree and 7 = strongly agree, please indicate how much you agree or disagree with each statement’. Agreement with items 1–38 indicates a tendency to think about pain in a body-centric way. Agreement with items 39–60 indicates a tendency to think about pain in a mind-centric way. The second column in the table reports mean responses. The third column reports mean responses after inverting the scale for items 1–38. This inversion allows the results to be plotted on a single scale where 1 = strong agreement with a bodily view and 7 = strong agreement with a mind-centric view. The data provided are for 69 participants (Table 2).

**Table 2:** List of 60 experimental items with mean responses and standard deviation (SD)

Experimental item	Mean response before inverting (1 = strongly disagree; 7 = strongly agree)	Mean response after inverting (1 = strongly body-centric; 7 = strongly mind-centric)	SD
1. When someone is in pain they can sometimes get so wrapped up in other things (like reading a good book or trying to do a puzzle) that they are not aware of their pain for a time, even though the pain still exists	5.14	2.86	1.31
2. When someone gets distracted from pain, the pain is still there, the person just doesn’t notice it	5.48	2.52	1.16
3. It is possible for a person to have a pain that they don’t feel for a period of time	4.64	3.36	1.61
4. It is possible for a person to have a pain that doesn’t hurt for a period of time	4.96	3.04	1.44
5. It is possible to have a pain that is not felt	4.32	3.68	1.61
6. Pains are properties of body parts—just as someone can have one leg that is slightly longer than their other one but not know this, so they can have one leg that has a pain and one that does not have a pain, but not know this	4.06	3.94	1.68
7. Someone can have pain in their leg without knowing it	4.12	3.88	1.60
8. Towards the end of a match, a footballer pulls a muscle but doesn’t feel any pain until the game is finished. The footballer had pain all along, but was too wrapped up in the game to notice	5.26	2.74	1.43
9. In rare conditions where two people share a body part (so-called ‘conjoined twin’ cases), if that shared body part is injured and one twin has pain then the other twin will also have pain	4.12	3.88	1.45
10. It is possible that two people can share a body part, as in ‘conjoined twin’ cases. Imagine that Melinda and Mary-Lou have been born conjoined at the waist and have a shared leg. One day their shared leg is damaged (they trip and cut their leg on a stone). Melinda says there is a pain in the leg, while Mary-Lou says there is no pain. In this case either Melinda or Mary-Lou must be wrong	3.17	4.83	1.64
11. Melinda and Mary-Lou are conjoined twins who share a leg. They stub a toe on the foot of the shared leg. Melinda says “ouch”, Mary-Lou doesn’t seem to notice the stubbed toe. In this case, both twins have pain	3.80	4.20	1.77
12. It is possible that someone can be wrong when they feel like they are in pain (e.g. because what they are experiencing is actually some other sensation, like heat or cold)	4.61	3.39	1.57
13. It is possible that someone can be wrong when they feel like they are in pain	4.16	3.84	1.66
14. It is possible to hallucinate pain, i.e. someone could feel like they are experiencing pain when they do not have any pain	4.70	3.30	1.49
15. When someone says “I am in pain” they are talking about a property of their body	4.77	3.23	1.43
16. When someone has a pain in their finger the pain exists in their finger	4.86	3.14	1.57
17. Pains are always telling us something about the state of our bodies	5.26	2.74	1.41
18. When somebody tells you they have pain in their finger, they are always telling you about a problem with their finger	4.57	3.43	1.72
19. Pain in a limb is always the result of some change to that limb (e.g. tissue damage or injury to that body part)	4.51	3.49	1.53
20. Pain is always the result of some change to one’s body (e.g. tissue damage or injury to some part of the body)	4.64	3.36	1.83
21. Pain is always the result of some bodily disturbance	4.70	3.30	1.66
22. If we feel pain, some part of our body must be damaged	4.32	3.68	1.69
23. It is possible that pain killers mask the experience of pain, even though the pain itself still exists	5.52	2.48	1.38
24. The only way to get rid of a pain is by curing the damage to the body to which the pain relates	4.45	3.55	1.72
25. Pain does not go away until the injury that causes it goes away	4.38	3.62	1.51
26. It is not uncommon for someone who has gone through a serious emotional event, such as the death of a loved one or the loss of a partner, to say that the event caused them pain. However this kind of talk can only be metaphorical because genuine pain is always caused by bodily damage	3.99	4.01	1.93
27. Imagine a creature that has no conscious awareness. If this creature suffers a sufficiently bad bodily injury, it will have pain, even though the creature will never be aware of this	4.14	3.86	1.79
28. A worm is able to have pain	4.22	3.78	1.60
29. Fish have pain in the same way that people do	3.84	4.16	1.72
30. The amount of pain someone has is directly related to the amount of tissue damage that has occurred	4.52	3.48	1.63
31. More tissue damage equals more pain	5.06	2.94	1.48
32. More tissue damage always equals more pain	4.51	3.49	1.54
33. It is possible to accurately measure someone’s pain without asking them	3.39	4.61	1.80
34. Objectively measuring the severity of someone’s injury is a more accurate way to know how much pain they are in than asking them	4.39	3.61	1.59
35. It is hard to trust people’s reports of their own pain	3.88	4.12	1.77
36. Bob claims he has pain in his lower back. His doctor has run every possible test and can’t find anything wrong. Bob is probably mistaken about having pain	3.13	4.87	1.81
37. Some people who claim they are in pain are actually imagining their pain	3.71	4.29	1.57
38. Two people who experience the same injury will have the same pain	2.86	5.14	1.71
39. It is not possible for a pain to exist if the subject is never aware of it	4.14	4.14	1.62
40. Someone can’t be in pain if they are not aware of it	4.16	4.16	1.71
41. All pains are felt	4.88	4.88	1.81
42. All pains hurt	5.32	5.32	1.54
43. While you have a pain, you will always be feeling that pain	4.35	4.35	1.88
44. If a person stops feeling a pain, they do not have the pain anymore	4.25	4.25	1.59
45. It is possible for someone to be seriously injured without that injury ever causing pain	4.20	4.20	1.74
46. Pain is a property of the mind	4.55	4.55	1.60
47. Pain is part of how we experience things that happen in the body	5.42	5.42	1.27
48. A person cannot be wrong about whether or not they have pain	4.36	4.36	1.75
49. If a pain killer gets rid of the experience of pain for a subject, then that person’s pain is gone (at least for the time that the medication is effective)	4.93	4.93	1.65
50. How much pain someone is in can sometimes depend on how they are thinking about their pain	5.12	5.12	1.31
51. It is possible to overcome pain mentally	4.57	4.57	1.66
52. If someone who is in pain takes pain medication and after a time they no longer feel any pain then they do not have any pain at that time	4.33	4.33	1.82
53. Two people who undergo exactly the same damage to the body (e.g. they suffer burns of exactly the same degree on matching areas of their hands) may have different levels of pain, to the extent that one of them may have pain from the injury while the other doesn’t	5.13	5.13	1.45
54. The International Association for the Study of Pain is right to define pain as “always a psychological state”, even if it is caused by an injury	4.30	4.30	1.48
55. Social exclusion or romantic loss can lead to pain	5.30	5.30	1.54
56. Although pain evolved to warn us about bodily damage, sometimes pain doesn’t perform any useful role in informing us about the state of our bodies	4.42	4.42	1.50
57. Someone can be talking about the same pain if they say: “I had a pain in my shoulder but now that pain has moved to my lower back”	4.80	4.80	1.41
58. A friend felt pain in their shoulder but now feels it in their back. Nevertheless, they identify it as “the same pain”. This is the same pain, even though it has changed locations	4.61	4.61	1.63
59. Two people who suffer an injury involving exactly the same amount of tissue damage may nevertheless have different amounts of pain	5.90	5.90	1.14
60. Thoughts and feelings can influence the amount of pain someone has	5.49	5.49	1.36

## Footnotes

1 Of course, holding that pain is a psychological state is not to deny that treatment often targets the bodily damage thought to be causing the pain or that clinicians’ focus is often on the somatic aspects of a patient’s pain.

2 We note that there are philosophical views which do not acknowledge the putative dichotomy between mental and bodily aspects of pain. Such a view is perhaps most famously associated with Ludwig Wittgenstein [8], but it also seems to underpin the work of John McDowell, who writes, for instance: What warrants the assertion that another person is in pain, on one of the relevant occasions, is the detectable obtaining of the circumstance of that person’s being in pain: an instance of a kind of circumstance—another person’s being in pain—that is available to awareness, in its own right and not merely through behavioural proxies. [9, p. 304]

3 The term ‘polyeidic’ was first introduced in Emma Borg et al. [10] as a technical label for the theory that the ordinary concept of pain is made up of distinct elements or dimensions.

4 Some descriptions of pain are relatively neutral and could, in principle, be understood in either mind-centric or body-centric terms. For example, utterances concerning amounts of pain (e.g., ‘I’m in a lot of pain’) could potentially be understood in either way—as concerning the intensity or severity of a feeling or the extent or degree of bodily damage. Likewise, utterances in which someone is described as being ‘in pain’ would seem to be compatible with mental or bodily ways of thinking. Justin Sytsma has suggested that he might want to pursue a different kind of route, treating ‘in pain’ as idiomatic (personal communication, October 2020). However, we will not consider this idea further here.

5 There are significant challenges in teasing apart the effects of individuals’ priors and the effects of context. As will become apparent below, one way in which we have sought to do so is by probing intuitions on short, simple statements, as opposed to the kind of rich scenarios we and others used in earlier empirical work, as this reduces the complexity of the context. We are grateful to an anonymous reviewer for stressing the relevance of this.

6 Empirical findings offered in support of the polyeidic view, to be discussed below, indicate that ordinary people do sometimes think of pains as conscious feelings—a conception that can be seen to lie at one end of a spectrum ranging from mind-centric to body-centric thinking about pain. Whether or not people also sometimes think of pains as unconscious mental properties is a question we leave for future research.

7 As noted by Reuter and Sytsma, agreement with this claim may indicate only that someone believes pains must hurt *at some point*, not that they must always hurt [6, p. 1795].

8 A related claim is that pain is *incorrigible*—which is to say that one cannot be wrong about whether or not one has it. Whether or not people hold this view may depend on whether conscious experience, in general, is thought to be incorrigible. For further philosophical discussion of the (in)corrigibility of conscious experience, see [17–19].

9 We are assuming here that people may think the intensity of a feeling can be influenced by other thoughts and feelings but that they will not think the extent of tissue damage can be influenced in this way. The interpretation becomes less clear, though, on an alternative version of the bodily view, such as one which appeals to nociceptive states rather than bodily damage/injury (see footnote 12 below).

10 We note that some of the statements put forward for probing people’s views of pain include modal language, raising questions about exactly how this is being understood by experimental participants. However, this is beyond the scope of the current research, which takes a comparable approach to that of previous empirical studies.

11 We set aside what are clearly tricky cases for advocates of the bodily view, concerning ‘referred’ pain or ‘phantom limb’ pain, where bodily damage is not located at the place where the damage is felt to have occurred. For further discussion of how such phenomena might be accommodated by the bodily conception of pain, see [10, pp. 32–33; 20, pp. 157–158].

12 While laypeople are unlikely to have sophisticated nociceptive conceptions of pain, they may think of pain in terms of nerve activity of some kind (we are grateful to an anonymous reviewer for stressing this point). While this is right, and would present a version of the bodily view that avoids a number of the worries related to the connection between pain and bodily injury to be discussed below, the well-attested point that pain is not simply a ‘direct readout of nociceptive input’, as Katja Wiech puts it [21], will cause difficulty for this view as well. For pain to exist, it might be thought that nerve activity must cause an experience of pain in the subject. In that case, the approach collapses into a mental view.

13 A more moderate version of this claim would be that pains need not be continuously felt in order to exist, but that they must be felt *at some point*. In other words, a pain can exist unfelt for a period of time, but it cannot exist if it is never felt. However, we will continue to restrict our focus here to the bodily conception discussed by Reuter and Sytsma, which makes the stronger claim that pain can exist even if it is never felt; for further discussion, see [6].

14 And such a feeling is clearly not considered to be incorrigible (see footnote 8 above).

15 Again, this is assuming that tissue damage is impervious to how one thinks or feels. We note, though, that if ‘overcome’—as in ‘It is possible to overcome pain mentally’—is read as something like ‘cope with’ rather than ‘eliminate’, someone operating with a bodily conception could perhaps accept the statement after all.

16 In total, seventy participants were recruited through Amazon Mechanical Turk and paid $2 for their participation. One participant was removed from the analysis for giving very similar responses across all experimental items, leaving sixty-nine participants—62.32% male, 36.23% female, 1.45% other (e.g., nonbinary). The average age was 37.04 years, with an age range from 21 to 69 years. Participants were presented with sixty statements, distributed quasi-randomly across eight blocks, with each block presented on a separate screen. Within each block, the order of the statements was randomised. Participants were required to answer all questions in the block before they could advance. They could not go back to review or change answers in previous blocks.

17 Unfortunately, we do not have space here for a detailed discussion of exactly why certain items seem to elicit generally mind-centric responses, while others seem to elicit generally body-centric responses. However, see the subsection on individual differences below for a little further discussion.

18 One might worry here that participants were simply agreeing with the statements presented to them, regardless of whether agreement would indicate mind-centricity or body-centricity. However, several of the experimental items elicited general disagreement (e.g., ‘Two people who experience the same injury will have the same pain’). Interestingly, in all such cases, disagreement indicated mind*-*centricity.

19 Another possibility is that the experimental items we selected, when taken as a whole, failed to reveal individuals’ pain priors. In the next stage of research, the battery of items will be further refined, to ensure that they are best placed to do so.

20 Interestingly, Reuter and Sytsma [6] find that only a minority of participants choose ‘all pains hurt’ when they are also presented with the following options: ‘most pains hurt’, ‘some pains hurt’, ‘no pains hurt’ (though ‘all pains hurt’ was the most common answer among them). While their findings may seem to conflict with ours, they remain consistent with our broader point—namely, that people’s views about pain can be affected by various contextual factors.
