# Correction to: Pain priors, polyeidism, and predictive power: a preliminary investigation into individual differences in ordinary thought about pain

**DOI:** 10.1007/s11017-022-09564-5

**Published:** 2022-03-21

**Authors:** Emma Borg, Sarah A. Fisher, Nat Hansen, Richard Harrison, Deepak Ravindran, Tim V. Salomons, Harriet Wilkinson

**Affiliations:** 1 University of Reading, Reading, UK; 2 University of Vienna, Vienna, Austria; 3 Royal Berkshire NHS Foundation Trust, Reading, UK; 4 Queen’s University, Kingston, ON Canada

## Correction to: Theoretical Medicine and Bioethics (2021) 42:113–135 10.1007/s11017-021-09552-1

The article “Pain priors, polyeidism, and predictive power: a preliminary investigation into individual differences in ordinary thought about pain”, written by “Emma Borg· Sarah A. Fisher· Nat Hansen · Richard Harrison· Deepak Ravindran· Tim V. Salomons· Harriet Wilkinson”, was originally published Online First without Open Access. After publication in volume 42, issue 3–4, page 113–135 the author decided to opt for Open Choice and to make the article an Open Access publication. Therefore, the copyright of the article has been changed to © The Author(s) 2022 and the article is forthwith distributed under a Creative Commons Attribution 4.0 International License, which permits use, sharing, adaptation, distribution and reproduction in any medium or format, as long as you give appropriate credit to the original author(s) and the source, provide a link to the Creative Commons licence, and indicate if changes were made. The images or other third party material in this article are included in the article’s Creative Commons licence, unless indicated otherwise in a credit line to the material. If material is not included in the article’s Creative Commons licence and your intended use is not permitted by statutory regulation or exceeds the permitted use, you will need to obtain permission directly from the copyright holder. To view a copy of this licence, visit https://creativecommons.org/ licenses/by/4.0. The original article has been corrected.

